# The Role of Nutritional Therapy in Alcoholic Liver Disease

**Published:** 2006

**Authors:** Christopher M. Griffith, Steven Schenker

**Affiliations:** Christopher M. Griffith, M.D., is a fellow of Gastroenterology and Steven Schenker, M.D., is professor of Medicine and Pharmacology, both at the University of Texas Health Science Center at San Antonio, San Antonio, Texas

**Keywords:** Ethanol metabolism, heavy alcohol use, alcoholic liver disease, alcoholic fatty liver, alcoholic hepatitis, fibrosis, alcoholic cirrhosis, gut, infection, anorexia, hepatic encephalopathy, nutrition, malnutrition, nutritional therapy, S-Adenosylmethionine (SAM), antioxidants, milk thistle, silymaryin, phosphatidylcholine, dietary fat, vitamins

## Abstract

Alcoholic liver disease (ALD) evolves through various stages, and malnutrition correlates with the severity of ALD. Poor nutrition is caused both by the substitution of calories from alcohol for calories from food and by the malabsorption and maldigestion of various nutrients attributed to ALD. The only established therapy for ALD consists of abstinence from alcohol. Sufficient nutritional repletion coupled with appropriate supportive treatment modalities may be effective in reducing complications associated with ALD—particularly infection. Nutrition makes a significant positive contribution in the treatment of ALD, especially in selected malnourished patients.

The study of malnutrition in patients with alcoholic liver disease (ALD) is based on several general concepts and observations. Researchers and clinicians previously believed that malnutrition was the primary cause of liver injury in ALD rather than the consequence of excessive alcohol consumption. This view was based on the prevalence of malnutrition in alcoholics and those with clinical evidence of liver (i.e., hepatic) dysfunction resulting from alcohol consumption (Mendenhall et al. 1984). It now is widely accepted that the quantity and duration of alcohol consumption are the principal agents in the development of alcoholic liver injury. This is based on animal and human data showing that ALD can develop in well-nourished individuals who consume large amounts of alcohol (Mezey 1991). However, a great deal of variability exists regarding the individual development of progressive alcoholic liver injury. Although more than 90 percent of people with excessive alcohol consumption will develop fatty liver (defined as greater than 5 percent fat in the liver), only up to 35 percent will develop inflammation of the liver caused by alcohol (i.e., alcoholic hepatitis) and only 20 percent will progress to scarring of the liver (i.e., cirrhosis) (McCullough and O’Connor 1998). Clearly, other risk factors, including genetic predisposition, obesity, concomitant viral hepatitis infection, and poor nutrition, may contribute variably to the development of ALD.

Indeed, in a large study of hospitalized patients with varying severity of ALD, malnutrition (especially the type caused by deficient protein and calories) was closely associated (although not necessarily causal) with the severity of liver injury (Mendenhall et al. 1984). All patients with clinical evidence of ALD (regardless of severity) exhibited some features of malnutrition. With regard to the possible value of nutritional therapy, it would seem logical that patients with more severe deficits would benefit more, although convincing proof of this, to our knowledge, is not available.

This article reviews the various forms of liver injury; the basis for and role of malnutrition in ALD, including the harmful effects of the products of alcohol metabolism; evidence for the benefits of nutrition; and special considerations for nutritional therapy in ALD.

## Diversity of Liver Injury

Alcoholic liver injury is known to evolve through various stages. Patients exhibit a variety of clinical symptoms and signs in liver histology (as reviewed in Dasarathy and McCullough 2003). As previously mentioned, almost everyone with heavy alcohol consumption develops fatty liver. This often is a rather benign disorder and considered reversible upon cessation of alcohol intake. In some cases, continued drinking may result in the development of alcoholic hepatitis, which may—and often does—end in severe clinical disease. The severity of disease and liver dysfunction correlates with an increasing short-term mortality (as reviewed in Dasarathy and McCullough 2003). It is in this acutely diseased group that optimal nutritional therapy might have the most impact. Continued drinking in this group of patients may lead to the development of excess scar tissue in the liver (i.e., fibrosis) and the subsequent anatomical changes of cirrhosis. Cirrhosis is considered a late stage of the disease, clinically manifested by progressive liver dysfunction with associated yellowing of skin and whites of the eyes (i.e., jaundice)—caused by decreased liver clearance of bilirubin, fluid accumulation in the abdomen (i.e., ascites), and impaired brain function caused by the accumulation of ammonia in the brain tissues (i.e., encephalopathy) (as reviewed in Dasarathy and McCullough 2003).

These three disorders may occur separately or often in association with each other. In many instances, these stages of liver injury may be difficult to distinguish either clinically or by laboratory measures of liver dysfunction (as reviewed in Dasarathy and McCullough 2003). Prior studies regarding nutritional therapy in ALD often did not differentiate between these various types of liver disease, complicating researchers’ abilities to assess the true benefit of nutritional therapy. This has been a problem in the assessment of such data in the past as well as for the present authors.

## Basis for Malnutrition in ALD

The signs and symptoms of nutritional deficits in ALD patients have been well characterized (Mendenhall et al. 1984; Mezey 1991; Schenker and Halff 1993; Nompleggi and Bonkovsky 1994; Hirsh et al. 1999) and include muscle wasting, decreased lean body mass, various vitamin deficiencies, and decreased measurable serum proteins. A complete description of specific nutritional deficits is beyond the scope of this review; however, it is important to consider the factors that contribute to malnutrition in individuals with ALD, as they may have an influence on the administration of nutritional therapy. There are many reasons for the deficits and abnormalities that occur as a result of malnutrition. These are outlined below and include decreased dietary intake and poor absorption and digestion of nutrients.

### Decreased Food Intake

People with ALD will substitute calories from food with calories from alcohol. It has been shown in patients with ALD that calories from alcohol may contribute more than 50 percent of their total calories, with calories from protein comprising only 6 to 10 percent (Mendenhall et al. 1984). In addition, the proportion of calories from alcohol appears to increase, whereas those from food decrease with escalating liver dysfunction (Mezey 1991). This increase in alcohol calories and decrease in food calories may be partially explained by the diversion of funds from the purchase of food to that of alcohol; however, hospitalized patients with ALD given adequate access to nutrition still demonstrate decreased ingestion of nonalcohol calories (Schenker and Halff 1993). This decreased desire for food (i.e., anorexia) also correlates with the severity of liver injury (Hirsh et al. 1999; Mendenhall et al. 1995). Anorexia is pervasive in ALD and is a key reason for decreased dietary intake of nonalcohol calories (Mezey 1991).

### Poor Absorption and Digestion of Nutrients

Another factor contributing to poor nutrition in patients with ALD is the malabsorption and maldigestion of various nutrients from the gut (Mezey 1991; Schenker and Halff 1993). This may relate to the impaired output of bile from the liver, resulting in decreased absorption of fat and fat-soluble vitamins. The possibility of concomitant pancreatitis causing decreased output of enzymes necessary for absorption of fats and proteins also can occur with alcohol abuse. Moreover, there may be a direct effect of alcohol on the gut itself. Alcohol has been demonstrated to decrease the intestinal absorption of amino acids and various vitamins, particularly thiamine, folate, and B_12_ (Schenker and Halff 1993). Malabsorption also may occur through mechanical alterations in the gut. This may be attributed to increased intestinal swelling (i.e., edema) from a lack of structural proteins in the gut wall, decreased intestinal enzyme activity (i.e., lactase) required for carbohydrate digestion and absorption, and/or decreased absorption from increased swelling of the gut. The latter is attributed to increased pressure in the draining vein (i.e., portal vein) and lymphatic vessels connecting the intestines with the liver. The changes in intestinal digestion and absorption appear to reverse once the patient ceases drinking and starts to follow a normal diet, suggesting that nutritional replacement may be of special benefit (Mezey 1991).

Finally, the preferential metabolism of alcohol by the liver alters the metabolism of sugars (i.e., carbohydrates), fats (i.e., lipids), and proteins (i.e., amino acids/nitrogen) (Mezey 1991). Abnormal breakdown of fat results in the formation of triglycerides that are deposited in the liver, manifesting as fatty liver (Schenker and Halff 1993). Altered fat metabolism also may propagate fibrosis by increasing collagen formation (Schenker and Halff 1993). The altered functional mass of the liver from these increased deposits of fat and collagen may result in decreased stores of vitamins and carbohydrates. Glycogen, the storage form of carbohydrates in the liver, serves as an energy reserve for periods of increased energy need. Inadequate glycogen reserves cause the body to make use of other metabolic pathways for energy, such as the breakdown of muscle (Schenker and Halff 1993). This may explain the increased muscle wasting, increased nitrogen excretion in the stool, and negative nitrogen balance seen in patients with ALD. It is important to note that in healthy individuals these alternate pathways of energy usually are found only after periods of prolonged fasting or starvation. Patients with ALD, however, may begin to use alternative pathways after only an overnight fast, suggesting that the frequency of feeding is as important as the type of feeding (McCullough and O’Connor 1998).

The basis for nutritional deficits is summarized in Table 1. This subject also has been extensively discussed in another review (Schenker and Halff 1993), which can be referenced for more details.

## Role of Malnutrition in ALD

It is not precisely known how alcohol causes liver damage. The net effect of nutrition on the development of ALD may involve multiple factors, including free-radical damage and increased risk of infection.

### Free-Radical Damage

It has been observed that one of the toxic byproducts of alcohol metabolism (i.e., free radicals) may cause damage to the liver. Free-radical damage also occurs as a result of oxidation of lipids in cellular membranes and in the internal constituents (i.e., mitochondria) of the cell. This improper oxidation of fat can, in turn, lead to the increased fat deposition and fibrosis, described previously. Cell damage from this improper oxidation also results in an inflammatory response and the generation of various signaling chemicals (i.e., cytokines), which may further contribute to tissue injury (as reviewed in Dasarathy and McCullough 2003).

The liver has a built-in defense against harmful oxidation in glutathione, a compound that assists in the removal of the toxic byproducts of alcohol metabolism. Glutathione availability depends on the presence of certain amino acids that may be deficient in patients with ALD (Schenker and Halff 1993). One of these amino acids, S-adenosylmethionine (SAM), serves as a precursor for glutathione. Evidence exists that SAM is deficient in patients with ALD (Schenker and Halff 1993). Furthermore, membrane integrity depends on the availability of SAM, in addition to an ample supply of phospholipids. SAM is involved in the processing of phospholipids needed for cell membrane repair (Schenker and Halff 1993). It also has been observed that patients with ALD are deficient in vitamins (e.g., vitamin E) that may offer a protective effect as antioxidants (Schenker and Halff 1993).

### Increased Risk of Infection

Patients with ALD are at increased risk of infection (Schenker and Halff 1993; Hirsh et al. 1999) partly because alcohol directly suppresses the immune system but also because the altered protein metabolism observed in ALD results in decreased circulating antibodies needed to fight infection. The intestinal system also works as a barrier to prevent bacteria from inside the gut from crossing the intestinal wall and causing infection. In addition, the cells of the gut secrete an antibody that is unique to the gut and assists with fighting infection (Schenker and Halff 1993). There is evidence that the gut’s role as a barrier to infection decreases with poor nutrition, as well as with excess alcohol intake (Schenker and Halff 1993). Research in animals has shown that improved nutrition results in decreased translocation of bacterial organisms across the gut and a subsequent decrease in bacterial infections (Casafont et al. 1997). Generally, it is accepted that patients with advanced ALD have an increased risk of morbidity and mortality with surgical procedures. This increased risk may be related to higher infection risk and poor wound healing (Schenker and Halff 1993). Considering that chronic ALD, after cessation of drinking, is one of the more common indications for liver transplant (McCullough and O’Connor 1998), it is reasonable to suggest that improved nutrition in patients with ALD can improve the outcomes of surgical procedures by decreasing infection and improving wound healing.

In brief summary, the decreased ability to process hepatic fat, as well as the lack of key proteins and amino acids that may decrease the liver’s ability to neutralize the effects of free radicals generated by alcohol metabolism, may, in turn, result in damage to cell membranes and promote inflammation and cell death (i.e., necrosis). Such events also may lead to fibrosis and even cirrhosis. Thus, theoretically, improved nutrition could ameliorate these adverse events, enhance hepatic regeneration, and decrease the risk of infection, which is a common complication of advanced ALD and a leading cause of patient mortality.

## Evidence for Benefits of Nutrition

As shown in Table 2, there have been about 15 studies (14 controlled and 1 pilot study) evaluating the benefits of nutritional therapy in ALD. These studies have assessed the possible benefit of optimal nutrition in a variety of ways. They have evaluated the effects on nutritional status, liver tests, liver histology, and, most importantly, mortality.

### Variations in Studies

The studies reviewed here have wide variations, which make meaningful comparison difficult. For instance, there is a disparity in the severity of liver disease among study participants. Two studies (Diehl et al. 1985; Nasrallah and Galambos 1980) had no deaths and one had only 5 percent mortality (Naveau et al. 1986). When considering that mortality ranged from 12 to 47 percent in the other studies, this clearly suggests a less severe form of disease in the former groups. Additionally, the studies have variable patient populations with regard to stage of ALD. Four studies (Marchesini et al. 1990; Kearns et al. 1992; Cabre et al. 1990; Hirsch et al. 1993) primarily addressed alcoholic cirrhosis, whereas 11 (Diehl et al. 1985; Nasrallah and Galambos 1980; Naveau et al. 1986; Calvey et al. 1985; Cabre et al. 2000; Mezey et al. 1991; Achord 1987; Mendenhall et al. 1985; Bonkovsky et al. 1991*a*; Bonkovsky et al. 1991*b*; Simon and Galambos 1988) focused on patients with alcoholic hepatitis. However, a large number of patients in the studies involving alcoholic hepatitis also had underlying cirrhosis. Moreover, many of these studies utilized only a small number of patients, making meaningful statistical analysis difficult. The studies also differed in methods of nutrient administration (e.g., oral, feeding tube, or intravenous line) as well as compliance and length of treatment.

Patient compliance in the studies cited generally is not estimated and is therefore difficult to assess. Compliance is clearly likely to be better in patients given food intravenously or via a feeding tube. It also may be better in those with less severe ALD because they have a lesser degree of anorexia.

### Research Findings

Even with these stipulations, a number of conclusions emerge from the studies reviewed. In most studies, nutritional status appears to have been improved in patients given proper dietary intake, especially with regard to nitrogen balance. The best evidence of this was in the studies by Mezey and colleagues (1991) and Bonkovsky and colleagues (1991*a*,*b*). One study (Naveau et al. 1986) found no improvement in nutritional parameters. A better correlation with improvement in nutritional status was seen in those studies with more specific parameters of nutrition, such as visceral proteins (which are reactive to physiological changes in the body and therefore are valuable in identifying malnutrition), nitrogen balance, triceps skinfold thickness, and creatinine–height index (a ratio of a patient’s 24hour excretion of the waste product creatinine [which is related to muscle mass] and the expected normal creatinine excretion) (Mezey et al. 1991; Bonkovsky et al. 1991*a*,*b*}.

### Liver Tests

The bulk of the data available suggests that improved nutrition is reflected in specific improvements in liver tests such as serum albumin (a protein found in blood plasma), bilirubin (produced by the breakdown of hemoglobin and processed in the liver), transaminases (enzymes involved in breaking down amino acids), and blood clotting (i.e., coagulation). A few studies (Kearns et al. 1992; Mezey et al. 1991; Bonkovsky et al. 1991*a*,*b*) even demonstrated enhanced liver status as measured by more direct tests of liver function that involve hepatic uptake and clearance of various substances. Kearns and colleagues (1992) demonstrated improved clearance of antipyrine,^1^ whereas Bonkovsky and colleagues (1991*a*,*b*) showed no change in antipyrine clearance but did report improved results from the galactose elimination test^2^ with improved nutrition. Only two studies (Diehl et al. 1985; Achord 1987) evaluated the role of nutritional therapy on histology of the liver in ALD. There was a decrease in fat in one study (Diehl et al. 1985) and fibrosis in the other (Achord 1987) but no improvement in inflammation or necrosis (Diehl et al. 1985).

### Effect on Mortality

Despite improvement in nutritional parameters and tests of liver “function” in most studies, the majority did not demonstrate a change in mortality. Two studies did report improved survival with nutritional therapy. Cabre and colleagues (1990) studied patients with cirrhosis, the majority of whom had alcoholic cirrhosis, and demonstrated a decreased mortality rate during hospitalization with nutritional supplementation through a feeding tube. Nasrallah and Galambos (1980) were able to show improved survival in patients with alcoholic hepatitis with administration of intravenous amino acids.

Although a consistent improvement in survival has not been demonstrated in individual studies, the average composite mortality in 10 studies (Diehl et al. 1985; Nasrallah and Galambos 1980; Naveau et al. 1986; Calvey et al. 1985; Mezey et al. 1991; Achord 1987; Mendenhall et al. 1985; Bonkovsky et al. 1991*a*,*b*; Simon and Galambos 1988) involving amino acid therapy in alcoholic hepatitis was 9.6 percent in those treated compared with 17 percent in control subjects, suggesting a trend toward significantly improved mortality (McCullough and O’Connor 1998).

### Effect on ALD

Other recent evidence suggests that nutritional therapy may play a role in treatment for ALD. Cabre and colleagues (2000) compared the use of steroids with nutritional therapy in severe alcoholic hepatitis and noted an improved mortality in the steroid group during the first week, whereas the nutrition group had improved mortality over the long term. The latter appeared to be attributed to decreased risk of infection. Hirsch and colleagues (1993) also noted decreased hospitalization at 1 year in patients with alcoholic cirrhosis given supplemental nutrition. This decline also was attributed to a decreased rate of infection. A follow-up, uncontrolled study by the same authors demonstrated that improved nutritional status in patients with alcoholic cirrhosis resulted in improved cell-mediated immunity, perhaps explaining the reduced incidence of infection (Hirsh et al. 1999). An uncontrolled study by Alvarez and colleagues (2004), in which 13 patients with alcoholic hepatitis were treated with both steroids and nutritional therapy, resulted in a mortality of 15 percent at 1 year. This was a lower rate than expected (25 percent) in this group historically from treatment with either modality alone. Controlled data are needed to verify this finding.

#### Protein Tolerance

A review of the composite data also suggests that patients with ALD are able to tolerate proteins (i.e., amino acids/nitrogen) well (i.e., no encephalopathy), whether given orally or by feeding tube. The use of intravenous lines to provide nutrition increases the risk of infection but also is well tolerated. Somewhat surprisingly, even patients with severe ALD tolerate generous protein supplements without precipitation or worsening of hepatic encephalopathy. Protein is of theoretical concern in patients with impaired liver function because the intestinal breakdown of protein results in ammonia, which is normally processed by the liver to urea. The exact mechanism by which excess ammonia acts as a neurotoxin is not completely understood. It generally is accepted, however, to play a pivotal role in hepatic encephalopathy (Albrecht and Norenberg 2006). In brain cells, ammonia is converted into the presumably nontoxic amino acid glutamine (Albrecht and Norenberg 2006). A recent study proposes that glutamine actually may be neurotoxic, as surplus glutamine is converted back to ammonia and also exerts an individual effect in the development of hepatic encephalopathy and its cerebral complications (i.e., brain edema) (Albrecht and Norenberg 2006; Ferenci 1994).

As a consequence of impaired liver function, other avenues are needed to dispose of ammonia. One method is conversion of ammonia to glutamine by muscle. A recent study in rats with acute liver failure and resulting ammonia elevations demonstrated increased glutamine production by skeletal muscle. This suggests that preservation of muscle mass via better nutrition may be beneficial in preventing or ameliorating hepatic encephalopathy and its ensuing complications in patients with ALD (Chatauret et al. 2006). The best type of nutritional therapy remains uncertain; however, general guidelines and goals for nutritional support in patients with ALD have been proposed (Tables 3 and 4).

## Special Considerations

Several specific nutrients, outlined below, require further evaluation as nutritional therapy for ALD patients.

### SAM

S-Adenosylmethionine (SAM) is important in the synthesis of proteins and polyamines as well as of glutathione (Schenker and Halff 1993). As mentioned above, glutathione plays a major role in the removal of damaging free radicals. The enzyme that produces SAM (SAM synthetase) is deficient in patients with alcoholic cirrhosis, leading to decreased levels of SAM (Mato et al. 1999). Administration of SAM potentially could benefit ALD patients by improving levels of glutathione and decreasing the damage to liver cells caused by alcohol. SAM is relatively benign with few side effects (Rambaldi and Gluud 2001). Despite one study demonstrating improved survival with long-term administration of SAM (Mato et al. 1999), a Cochrane systematic review of eight randomized controlled trials did not demonstrate a survival advantage (Rambaldi and Gluud 2001). Therefore, this agent should not be used for treatment, pending further randomized controlled trials. Of note, the critical Cochrane review only considered one of the eight studies evaluated to be an adequate one.

### Antioxidants

As increased free radical formation appears to play a role in the development of ALD, there has been interest in the efficacy of antioxidants in the treatment of ALD. Metadoxine, an antioxidant with few known side effects, has been approved for use in the treatment of ALD in countries other than the United States. A controlled trial from Spain randomly assigned 136 alcoholics with fatty liver to receive either metadoxine or a placebo. After 3 months, both groups showed an overall improvement in markers of liver function; however, the metadoxine group demonstrated more improvement and a more rapid response to therapy (Caballeria et al. 1998). The metadoxine group also had a more substantial decrease in the amount of fat in the liver. Improvements were noted in those who continued drinking, although the recovery was better in those who were abstinent. Although the results are promising, it appears that further studies involving more advanced ALD are needed before recommending this as therapy for ALD patients.

### Milk Thistle (Silymaryin)

Milk thistle is a popular dietary herbal supplement used by many patients with chronic liver disease. Milk thistle primarily is composed of silymaryin (70 to 80 percent), which is its active ingredient (Ball and Kowdley 2005). Silymaryin appears to have antioxidant and toxin-reducing properties in the liver and has been shown to reduce liver fibrosis and enhance liver regeneration in animal studies (Lieber et al. 2003*a*). However, a systematic review and meta-analysis of studies involving milk thistle in patients with chronic liver disease, the majority of whom suffered from alcoholic cirrhosis, failed to detect a benefit in liver histology or mortality. The review did note that the use of milk thistle was safe and well tolerated (Jacobs et al. 2002). Another review on the use of milk thistle in ALD treatment concluded that studies on the use of milk thistle were too limited by poor quality and lack of standardization of the milk thistle preparation to recommend its use (Ball and Kowdley 2005).

### Phosphatidylcholine

Phosphatidylcholine is a soybean extract that also is a component of cell membranes. It theoretically is an attractive therapy because of its low side effects, antioxidant properties, and fibrosis-reducing effects (Lieber et al. 2003*b*). Studies with phosphatidylcholine in baboons demonstrated a reduction in hepatic fibrosis (Lieber et al. 1994). The positive results in animals led to a long-term, multicenter study in humans with chronic alcohol-induced liver disease. Patients with biopsy-proven early fibrosis attributed to alcohol were treated with phosphatidylcholine or a placebo for 2 years. Subsequent liver biopsies after treatment failed to demonstrate a beneficial effect on progression of hepatic fibrosis compared with patients taking a placebo (Lieber et al. 2003*b*). This may have been because of much lower than expected progression of fibrosis in the control group attributable to decreased alcohol intake in patients given supportive care. Thus, the projected statistical analysis could not be conducted. Further data are needed before this approach can be considered as therapy in this patient population.

### Dietary Fat

Fat has been suggested as a risk factor for the development and aggravation of ALD in animals and humans (Mezey 1998). More than 90 percent of heavy drinkers develop fatty liver (McCullough and O’Connor 1998). Continued alcohol ingestion in the presence of a fatty liver can lead to the development of fibrosis and cirrhosis. Countries with a diet high in saturated fats have a lower incidence of ALD despite equivalent alcohol ingestion (McCullough and O’Connor 1998). Research with animals also has shown that intake of increased saturated fat can reverse the changes of ALD despite continued alcohol intake (You et al. 2005). This may, in part, be attributed to less free-radical production from saturated fat. Moreover, a recent study (You et al. 2005) concluded that a diet high in saturated fats and alcohol stimulates the release of adiponectin from fat cells. It appears that adiponectin is responsible, at least partially, for the protective effect of saturated fats in animal models of ALD by its beneficial effect on hepatic fat metabolism and reduction in harmful cytokines. Diets high in saturated fats, however, are well known to increase the risk of cardiovascular disease in humans. Thus, studies involving nutritional supplementation of adiponectin as a therapy for ALD need to be undertaken before a diet high in saturated fats can be recommended.

### Vitamins

Vitamin deficiencies are common among people who consume excessive amounts of alcohol, even in the absence of liver disease. Deficits of vitamins A, B_1_ (thiamine), B_6_ (pyridoxine), and folic acid are of particular concern, as they not only are common but may exacerbate the detrimental effects of alcohol. Other vitamins that often are lacking in those with ALD include B_2_ (riboflavin), B_3_ (niacin), C, D, and E. Vitamin deficiencies in those with ALD are caused by a combination of decreased intake, absorption, and storage (Lieber 2003). In addition, alcohol often interferes with the conversion of vitamins to a metabolically active form: one example is vitamin A. The enzymes needed to convert vitamin A to its active form are part of the same family of enzymes that metabolize alcohol (Leevy and Moroianu 2005). The competition between vitamin A and alcohol results in decreased levels of active vitamin A.

Identification and restitution of vitamin deficiencies in patients with ALD is essential. This especially is true regarding vitamin B_1_ (thiamine), because thiamine deficiency can cause acute Wernicke’s encephalopathy, a degenerative brain disease that potentially is rapidly reversible (Lieber 2003). Replacement of vitamin A, however, is more complicated. Even standard recommended doses of vitamin A can become toxic with continued alcohol consumption. Moreover, it is difficult to estimate vitamin A levels because serum levels often do not reflect stores of vitamin A in the body. Many of the remaining vitamins (B complex, C, D, E, and folic acid) can be restored with amounts found in standard multivitamins (Lieber 2003).

Vitamin E deficiency and replacement is of special interest. As mentioned previously, there is an increased formation of free radicals in ALD (as reviewed in Dasarathy and McCullough 2003). Vitamin E is an antioxidant that prevents the degradation of lipids by oxidation (i.e., lipid peroxidation) and free radical formation (Traber and Sies 1996). It would appear logical that replacement of vitamin E potentially would be beneficial in treating ALD; however, one study (Mezey et al. 2004) that randomly assigned patients to receive vitamin E or a placebo did not demonstrate improvement in patients with mild to moderate alcoholic hepatitis.

## Conclusion

It is obvious that nutrition plays some part in ALD given the prevalence of malnutrition, especially of the protein–calorie type. The malnutrition usually is associated with disease seen in hospitalized patients and correlates with the severity of ALD. The primary, established therapy for ALD consists of abstinence from alcohol. Good nutrition improves nitrogen balance, may improve liver tests, and may decrease hepatic fat accumulation, but generally it does not enhance survival. This suggests that adequate nutrition is beneficial when administered with other forms of treatment but is not sufficient therapy by itself. It has been suggested that sufficient nutritional repletion coupled with other treatment modalities may be effective in reducing complications associated with ALD—particularly infection.

Optimal nutrition requires adequate protein calories and vitamins. Ideally, the patient should receive adequate nutrition orally or through a feeding tube. This may require a feeding tube that goes through the nose to the stomach (i.e., nasogastric) or, if that is not possible, intravenous nourishment. Administration of nutritional therapy (i.e., amino acids/nitrogen) is tolerated well with little adverse effect in patients with ALD. There apparently is no problem with the precipitation of hepatic encephalopathy. Specific nutrients (i.e., phosphatidylcholine, SAM, and saturated fat), although generally innocuous and well tolerated, require further evaluation. Overall, nutrition is not a panacea in the treatment of ALD, but it makes a significant positive contribution, especially in selected malnourished patients.

## Figures and Tables

**Table 1:** Basis for Nutritional Deficits in Alcoholic Liver Disease (ALD)

Decreased caloric intake	Results
• Anorexia	• Decreased calories, vitamins, and nutrients available for utilization
• Decreased ingestion of non-alcohol calories	
• Increases with severity of ALD	
**Decreased intestinal absorption/ digestion of nutrients**	
• Decreased bile excretion	• Decreased fat digestion and absorption of fat-soluble vitamins
• Decreased pancreatic function	• Decreased pancreatic enzymes for fat and protein digestion
• Altered intestinal integrity	• Decreased amino acid and vitamin absorption and digestion
• Decreased intestinal enzymes	• Decreased carbohydrate digestion
**Decreased processing and storage of nutrients**	
• Preferential metabolism of alcohol	• Abnormal processing of fats and sugars
• Abnormal oxidation of fat	• Fatty liver and increased collagen production
• Decreased functional liver mass	• Decreased energy stores and utilization of alternative pathways normally reserved for fasting (abnormal muscle breakdown and abnormal oxidation of fats)

SOURCE: Modified with permission from Mezey 1991 and Schenker and Halff 1993.

**Table 2:** Studies of Nutrition and Alcoholic Liver Disease (ALD)

Reference	ALD stage	# Pts	Route	Time (days)	Type of diet	Liver tests	Liver histology	Nutritional parameters	Mortality	Comments
Cabre et al. 2000	AH (80% AC)	71	Enteral vs. steroids	28	**Control:** 2,000 kcal/ 1g protein/kg/day oral diet + 40 mg/day prednisolone (actual intake not stated) **Treatment:** 2,000 kcal/72 g protein 31% as BCAA via NGT	Improved in both groups	Not assessed	Improved albumin in both; no change in anthropometrics	No over-all effect	Followed for 1 year; deaths earlier in NGT group (7 vs. 23 days); deaths in steroid group later; deaths in steroid group from infection
Mendenhal et al. 1985	AH	57	Oral and enteral	30	**Historic controls:** 2,500 kcal/day oral diet (actual ~2,300 kcal/day) **Treatment:** 2,500 kcal oral diet + 2,200 kcal/day BCAA supplement	No difference	Not assessed	Improved in treated group	No effect	Study not randomized; improved nutrition and nutritional parameters; mortality 17 to 21%
Calvey et al. 1985	AH	64	Oral (some IV)	21	**Control:** 1,800 to 2,400 kcal/70 to 100 g protein oral diet **Treatment:** control diet + 2,000 kcal supplement with 65 g conventional protein or 65 g protein as BCAA	No difference	Not assessed	Improved but no difference between groups	No effect	23% with HE not affected by diet; improved cutaneous energy; improved nitrogen balance; 32 to 43% mortality
Nasrallah and Galambos 1980	AH	35	Oral and IV	28	**Control:** 3,000 kcal/ 100 g protein oral diet (actual intake ~1,400 kcal/day) **Treatment:** control diet + 70 to 85 g AA IV (actual oral intake 1,500 kcal)	Improved in treated group	Not assessed	Not assessed; improved nitrogen balance	Improved (p <0.02)	Significant improvement in liver tests; improved nitrogen balance; 22% mortality in control subjects; small patient number
Diehl et al. 1985	AH	15	Oral and IV	30	**Control:** unrestricted diet (~3,000 kcal/day actual intake) **Treatment:** control diet + 53 g AA + 130 g glucose IV	No difference	Decreased fat but not inflammation or necrosis in treated group	Improved in treated group	N/A	Improved histology and nutrition; no mortality; patients less severely ill; small patient number
Achord 1987	AH	28	Oral and IV	21	**Control:** 2,700 kcal/day oral diet (actual intake ~1,200 kcal/day) **Treatment:** control diet + 42.5 g AA/day IV (actual oral ~1,200 kcal)	Improved in treated group (galactose elimination improved)	Decreased hyaline in treated group	Not assessed	No effect	Low oral intake in both; improved functional liver mass; improved liver tests; 7 to 21% mortality; small patient number not assessed
Simon and Galambos 1988	AH	34	Oral and IV	28	**Control:** 2,400 kcal/100 g protein oral diet + 3 cans Ensure (actual intake not recorded) **Treatment:** control diet + 70 g AA, 100 g glucose, 50 g lipid per day IV	No difference in moderate but improved in severe hepatitis in treated group	Not assessed	Not assessed	No effect	Improved liver tests in severe AH; stratification into moderate and severe AH decreased number for analysis
Mezey et al. 1991	AH	54	Oral and IV	30	**Control:** 2,000 kcal orally + 130 g glucose IV (actual oral intake ~1,400 kcal; total ~1,950) **Treatment:** control diet + 52 g AA/130 g glucose IV; (oral intake ~1,300 kcal; total ~2,300 kcal)	Improved in treated group (galactose elimination)	Not assessed	Improved in treated group	No effect	Improvement in liver tests and nutritional parameters; 1 month survival 19% controls and 21% treated; 2-year survival 40% in both
Bonkovsky et al. 1991	AH	39	Oral and IV	21	**Control:** oral 30 kcal and 1 g protein/kg IBW/day (actual intake ~1,800 kcal/day) **Treatment:** control diet + 70 g AA and 100 g glucose IV/day (~2,300 kcal oral + IV daily)	Trend toward improvement in treated group	Not assessed; decreased liver volume in treated group	Improved in treated group	No effect	No mortality during study; improved nutrition parameters; trend toward improvement in liver tests
Naveau et al. 1986	AC (23% AH)	40	Oral and IV	28	**Control:** 40 kcal/kg and 200 mg nitrogen/kg/day orally (actual intake ~2,100 kcal/day) **Treatment:** control diet as IV + offered oral diet; (actual oral intake ~1,100 kcal)	Improved in treated group	Not assessed	No significant improvement in treated group	No effect	Low difference in oral intake in treatment vs. control; only 5% total mortality; no improvement in nutrition parameters; some improvement in liver tests
Cabre et al. 1990	AC	35	Oral vs. enteral	23 to 25	**Control:** oral 2,200 kcal and 70 to 80 g protein/day; (40 g protein/day if HE); (actual intake ~1,320 kcal) **Treatment:** 2,115 kcal and 71 g protein as BCAA via FT	Improved in treated group (improved Child’s score)	Not assessed	Improved albumin in treated group; no difference in anthropometrics	Improved	Improved albumin and Child’s score in treatment; 12% mortality in treated vs. 47% in controls; no overall improvement in nutrition parameters
Kearns et al. 1992	AC	31	Oral with enteral support	28	**Control:** standard oral diet (actual not stated) **Treatment:** control diet + 1.5 g/kg/day supplement as casein; (reported 200% increase kcal intake vs. control)	Improved in treated group	Not assessed	Improved in treated group	No effect	Improved nitrogen balance and liver tests in treatment; 13% mortality in treated vs. 27% in control at 1 week, equal mortality at 8 weeks
Marchesni et al. 1990	AC	64	Oral	90	**Control:** 15 days 45 to 65 g protein then oral diet + 0.175 g/kg protein casein supplement **Treatment:** 15 days of 45 to 65 g protein then regular diet + 0.24 g/kg protein as BCAA supplement	Improved in treated group	Not assessed	Improved in both, but greater in treated group	Not assessed	Greater improvement in nitrogen balance in treatment; improved HE in treatment; crossover at 3 months with improvement in HE in those changed to treatment
Hirsch et al. 1993	AC	51	Oral	12 mo	**Control:** oral diet + placebo (actual ~1,580 kcal intake) **Treatment:** control diet + 1,000 kcal and 34 g protein/day orally (actual ~2,470 kcal intake)	Improved in both	Not assessed	Improved in treated group	No effect	Trend toward improved mortality in treatment; improvement due to decreased infection
Alvarez et al. 2004	AH	13	Enteral + steroids	22 (+/− 3.8)	**Control:** none **Treatment:** 2,000 kcal and 72 g protein orally/day + 40 mg prednisolone	Improved	Not assessed	Not assessed	15%^*^	Pilot study, not randomized, mortality less than expected from either historic treatment alone

AA = amino acids; AC = alcoholic cirrhosis; AH = alcoholic hepatitis; BCAA = branched chain amino acids; FT = feeding tube; HE = hepatic encephalopathy; IBW = ideal body weight; IV = intravenous; NGT = nasogastric tube; for comparison ^*^ = no control for comparison.

**Table 3:** Goals of Nutritional Supplementation in Chronic Liver Disease

Prevent or correct protein–calorie malnutrition Prevent or correct hepatic encephalopathy Aid hepatic healing and regeneration insofar as possible Improve quality of life Prolong life and improve prognosis after liver transplantation Control the costs and discomforts of therapy insofar as possible Avoid potential unwanted side effects of therapy, including encephalopathy, azotemia, electrolyte or water imbalance, aspiration, venous thrombosis or thrombophlebitis, and sepsis

SOURCE: Nompleggi and Bonkovsky 1994, with permission.

**Table 4:** Guidelines for Daily Dietary Feeding in Alcoholic Liver Disease (ALD)

**Protein = 1.0 to 1.5 kg body weight**
**Total calories = 1.2 to 1.4 times resting energy expenditure with a minimum of 30 kcal/kg body weight**
50 to 55% as carbohydrate (preferably as complex carbohydrates) 30 to 35% as fat (preferably high in unsaturated fat and with adequate essential fatty acids)
**Nutrition should be given enterally by voluntary oral intake and/or by small-bore feeding tube**
PPN is second choice TPN is last choice
**Salt and water intake should be adjusted for patient’s fluid volume and electrolyte status**
**Liberal multivitamins and minerals**
**Specialized BCAA-enriched supplements not usually necessary as most patients tolerate standard AA supplements**
Reserve BCAA formulations for patients who cannot tolerate the necessary amount of standard AA (which maintain nitrogen balance) without precipitating encephalopathy Avoid supplements providing only BCAA; they do not maintain nitrogen balance Conditionally essential AA as well as all essential AA are needed Conditionally essential AA are those that normally can be synthesized from other precursors but cannot be synthesized in cirrhotic patients. These include choline, cystine, taurine, and tyrosine

AA = amino acids; BCAA = branched chain amino acids; PPN = peripheral parenteral nutrition (nutrition is provided intravenously via a peripheral vein); TPN = total parenteral nutrition (nutrition is provided intravenously via a central vein, more concentrated than PPN).

SOURCE: McCullough and O’Connor 1998, with permission.

## Footnotes

1 Antipyrine metabolism is used as an index of liver function.

2 The galactose elimination test measures liver function.
